# Reviewing the literature, how systematic is systematic?

**DOI:** 10.1007/s11096-016-0288-3

**Published:** 2016-04-05

**Authors:** Katie MacLure, Vibhu Paudyal, Derek Stewart

**Affiliations:** School of Pharmacy and Life Sciences, Robert Gordon University, Aberdeen, AB10 7GJ Scotland, UK

**Keywords:** Critical appraisal, Data extraction, Literature, Review, Systematic review

## Abstract

*Introduction* Professor Archibald Cochrane, after whom the Cochrane Collaboration is named, was influential in promoting evidence-based clinical practice. He called for “relevant, valid research” to underpin all aspects of healthcare. Systematic reviews of the literature are regarded as a high quality source of cumulative evidence but it is unclear how truly systematic they, or other review articles, are or ‘how systematic is systematic?’ Today’s evidence-based review industry is a burgeoning mix of specialist terminology, collaborations and foundations, databases, portals, handbooks, tools, criteria and training courses. *Aim of the review* This study aims to identify uses and types of reviews, key issues in planning, conducting, reporting and critiquing reviews, and factors which limit claims to be systematic. *Method* A rapid review of review articles published in IJCP. *Results* This rapid review identified 17 review articles published in IJCP between 2010 and 2015 inclusive. It explored the use of different types of review article, the variation and widely available range of guidelines, checklists and criteria which, through systematic application, aim to promote best practice. It also identified common pitfalls in endeavouring to conduct reviews of the literature systematically. *Discussion* Although a limited set of IJCP reviews were identified, there is clear evidence of the variation in adoption and application of systematic methods. The burgeoning evidence industry offers the tools and guidelines required to conduct systematic reviews, and other types of review, systematically. This rapid review was limited to the database of one journal over a period of 6 years. Although this review was conducted systematically, it is not presented as a systematic review. *Conclusion* As a research community we have yet to fully engage with readily available guidelines and tools which would help to avoid the common pitfalls. Therefore the question remains, of not just IJCP but potentially all published reviews, ‘how systematic is systematic?’

## Impacts on practice

- Today’s evidence-based review industry is a burgeoning mix of specialist terminology, collaborations and foundations, databases, portals, handbooks, tools, criteria and training courses.
- Minimising bias is often aided by the independent application of recognised, standardised tools by more than one researcher conducting a review.
- As a research community we have yet to fully engage with readily available guidelines and tools which would help to avoid the common pitfalls in conducting reviews systematically.

## Introduction

Professor Archibald Cochrane, after whom the Cochrane Collaboration is named, was influential in promoting evidence-based clinical practice when he called for,

“the conscientious use of current best evidence in making decisions about the care of individual patients or the delivery of health services. Current best evidence is up-to-date information from relevant, valid research about the effects of different forms of health care” [1].

Today’s evidence-based review industry is a burgeoning mix of specialist terminology, collaborations and foundations, databases, portals, handbooks, tools, criteria and online training courses. Although systematic reviews and meta-analyses of randomised controlled trials (RCTs) are considered the gold standard within the hierarchy of evidence ahead, in order, of RCTs, cohort studies, case control, case series and case reports, qualitative studies, editorial articles and commentaries, pharmacists and researchers will benefit from an informed and critical approach to questioning the validity and applicability of published findings. Sir Ian Chalmers, celebrated health services researcher and one of the founders of the Cochrane Collaboration, favoured systematic reviews,

“because the results of a particular research study cannot be interpreted with any confidence unless they have been synthesised, systematically, with the results of all other relevant studies. Science is meant to be cumulative, but researchers usually don’t cumulate scientifically” [1].

Readers need to understand the differences between the many types of review as only well-conducted systematic reviews provide the highest level of evidence.

Grant and Booth’s [2] typology of 14 different types of review does not include either narrative reviews or reviews of reviews (critical, literature, mapping, meta-analysis, mixed methods, overview, qualitative systematic, rapid, scoping, state-of-the-art, systematic, systematic search and review, systematized, umbrella). It does, however, describe literature reviews as generic, which may or may not include systematic, comprehensive searching and quality assessment with narrative synthesis of findings often reported chronologically or thematically [2]. In contrast, systematic reviews are described as comprehensive and exhaustive, which “systematically search for, appraise and synthesise research evidence, often adhering to guidelines on the conduct” before reporting on, “what is known, recommendations for practice, what remains unknown, uncertainty around findings, recommendations for future research” [2]. So, while all literature reviews may be undertaken systematically, systematic reviews aim to appraise and synthesise evidence to answer specific review question(s) following a protocol written in advance. It is not unusual for reviews to be described by authors as systematic reviews when in actual fact they are narrative overviews of the available literature within a given field but do not aim to answer a specific review question. Therefore, it is especially important to understand and be able to critically appraise any review of the literature by posing the question, ‘how systematic is systematic?’ [1–6]?

## Aim of the review

This study aims to identify uses and types of reviews, key issues in planning, conducting, reporting and critiquing reviews, and factors which limit claims to be systematic in practice.

## Method

A rapid review of the IJCP publications database (2010–2015) was conducted to identify articles with ‘review’ in the title. Data were extracted to demonstrate the range of review types presenting as systematic in title, systematic in method or—through the application of a range of guidelines, criteria and tools—systematic in conduct to provide evidence in answering the challenge ‘how systematic is systematic?’.

## Results

Seventeen articles were identified from their title as being forms of review of which ten were systematic reviews, three were reviews of reviews, two were narrative reviews and two were literature reviews (Table 1) [7–23].

**Table 1:** Results of rapid review of reviews published in IJCP (2010–2015)

Year	Title	Type of review^a^	Clear review question?	Protocol referenced?	Method described as ‘systematic’?	Evidence of team approach?	Clear search strategy?	Uses recognised checklists?
2011	Literature review on the structure and operation of Pharmacy and Therapeutics Committees [7]	L	Y	N	N	Y	Y	N
2012	An analysis of quality of systematic reviews on pharmacist health interventions [8]	R	Y	N	N	Y	Y	AMSTAR
2014	Evidence for compliance with long-term medication: a systematic review of randomised controlled trials [9]	S	Y	N	Y	N	N	N
2014	Adverse drug reactions associated with asthma medications in children: systematic review of clinical trials [10]	S	Y	N	Only in the abstract and title	Y	Y	PRISMA
2014	Look alike/sound alike drugs: a literature review on causes and solutions [11]	L	Y	N	No, justification given as ‘the real difficulty to have sufficient data on specific outcomes to perform a systematic review or a meta-analysis, it was designed a specific methodology to make a narrative review of the articles collected’	Y	Y	N
2014	Systematic review of efficacy and safety of pemetrexed in non-small-cell-lung cancer [12]	S	Y	N	Yes, but also as literature review	Y	Y	CASP
2014	Systematic review of severe acute liver injury caused by terbinafine [13]	S	Y	N	No, only in the title	Only at quality assessment	N	Mentions PRISMA, RevMan and Jadad Scale
2014	A systematic review of the literature on medication wastage: an exploration of causative factors and effect of interventions [14]	S	Y	Yes, but not published	Yes, ‘Systematic reviews differ from more traditional, narrative reviews, collating and synthesising evidence according to pre-specified eligibility criteria in order to address specific research questions, while minimizing bias by adhering to explicit, systematic methods’	Y	Y	CASP
2014	Assessment of pharmacist-led patient counseling in randomized controlled trials: a systematic review [15]	S	Y	N	Y	Y	N	PRISMA, Jadad Scale
2014	Design and comprehensibility of over-the-counter product labels and leaflets: a narrative review [16]	N	Y	N	No, it was considered and rejected	‘The choice of conducting a narrative review, as opposed to a systematic review of the literature, has allowed for a wider scope of literature to be reviewed. However, it is important to acknowledge that in future, a systematic review to specifically focus on certain areas of OTC medicine information design and comprehensibility research may be considered’		N
2014	Economic evaluations of clinical pharmacist interventions on hospital inpatients: a systematic review of recent literature [17]	S	Y	N	Y	Y	Y	CHEERS statement
2014	Evaluation of pharmacist care for patients with chronic obstructive pulmonary disease: a systematic review and meta-analysis [18]	S	No, varies between evaluation, influence and impact of pharmacist care	N	Only in the title and occasionally in the text	Y	No, example search not included	GradePro, RevMan
2014	Methoxy polyethylene glycol-epoetin beta versus darbepoetin alfa for anemia in non-dialysis-dependent CKD: a systematic review [19]	S	Y	Yes, but not published	Y	Y	Y	PRISMA, Jadad Scale
2015	A systematic review of community pharmacist therapeutic knowledge of dietary supplements [20]	S	Y	Yes, but not published. Also incorrectly referenced in text.	Y	Y	Y	N
2015	Pharmacists medicines-related interventions for people with intellectual disabilities: a narrative review [21]	N	Y	N	Y	Y	Y	N
2015	Effectiveness of clinical pharmacy services: an overview of systematic reviews (2000–2010) [22]	R	Y	N	No, this review of systematic reviews follows ‘the Cochrane Collaboration recommendations and the PRISMA statement’ but does not describe itself as ‘systematic’	Y	Y	R-AMSTAR, DEPICT, PRISMA
2015	The relevance of systematic reviews on pharmaceutical policy to low- and middle-income countries [23]	R	Y	N	Y	Y	Y	N

^a^Type of review: *L* literature, *R* review of systematic reviews, *N* narrative, *S* systematic review

## Uses: the types and purposes of reviews

As shown by the 17 identified IJCP review articles (Table 1), reviews cover the spectrum of healthcare including drugs, technologies, pharmacy practice and practice of the wider, often multidisciplinary, health and social care team. The articles for inclusion in a systematic review may be reporting primary data based on qualitative (often interviews or focus groups), quantitative (surveys or RCTs), mixed methods or based on secondary data reported cumulatively in systematic reviews (a review of reviews). The systematic review is typically written in a narrative (descriptive) form with accompanying tables describing the included, cumulative evidence but other variations include critical, rapid, literature, meta-narrative scoping and reviews of systematic reviews [2]. The Centre for Review and Dissemination at the University of York recommends checking for existing systematic reviews already registered or conducted (DARE, CDSR, NICE, NIHR HTA, EPPI, NGC, SIGN)^1^ while also scoping the breadth of the relevant literature (MEDLINE, EMBASE, CINAHL, IPA, ERIC) [24]. If an existing systematic review is found, is it clearly relevant, valid and of good quality; is there a clear need for it to be updated or extended?

Pharmacists can benefit from gaining experience of considering where application of high quality relevant findings can bridge the evidence gaps and so promote safety, efficacy and effectiveness of evidence-based pharmaceutical interventions.

The reasons for conducting a systematic review are many and varied including to:

- identify, evaluate and interpret available research evidence relevant to a particular topic;
- identify effective and ineffective healthcare interventions;
- help inform practice and policy by providing integrated and unbiased evidence on which to base decisions; and
- identify gaps in the literature to inform future studies [1].

As systematic reviews, “aim to minimize bias by using explicit, systematic methods”, a review protocol is developed, and often registered or published, which sets out the methods to be used,

“including decisions about the review question, inclusion criteria, search strategy, study selection, data extraction, quality assessment, data synthesis and plans for dissemination” [24].

## Elements of a systematic review protocol

A well formulated protocol is an essential component of a systematic review as it guides the course of the whole review process and differentiates from other types of reviews. Starting from a specific and targeted review question, then outlining resources for gathering all relevant studies, systematic review protocols enable predetermined and explicit method of undertaking a systematic review through to write up and dissemination. The systematic review protocol aims to describe the plans to be followed in the review, designed to minimise bias by explicitly stating a priori hypotheses and methods, without advance knowledge of results [24–26]. Of the 17 review articles identified (Table 1), only three mention a predetermined review protocol but none published a protocol. Depending on the healthcare subject area, the protocol may be accepted for registration with institutions such as one of fifty-three Cochrane Review Groups or the Joanna Briggs Institute (JBI)—both of whom require approval of a title registration form prior to development of the review protocol—or PROSPERO which is associated with CRD [27–29]. But registration of a protocol necessitates a commitment to maintaining an auditable log of any changes. Some, including Cochrane, ask the authors to commit to regular updates which the resourceful reviewer will facilitate by saving searches in key databases and setting up email alerts from key sources. Some journals, for example BioMed Central or the Systematic Review Journal, also accept systematic review protocols for publication [30, 31]. The recent publication of PRISMA-P (2015) is a welcome additional guide to writing systematic review protocols [32]. Moher et al. [32] suggest “peer reviewers and editors can use the guidance to gauge the completeness and transparency of a systematic review protocol submitted for publication”. The PRISMA-P checklist consisting of 17 items (Table 2) was developed through consensus methods guided by an international steering group of experts in systematic reviews drawing on best practice from CDSR, PROSPERO, AHRQ and more [27, 29, 40].

**Table 2:** Extracts from the PRISMA-P 2015 checklist [32]

Section/topic	#	Item
Title	1	Identification Update
Registration	2	Name of the registry
Authors	3	Contact Contributions
Amendments	4	State plan for documenting
Support	5	Sources Sponsor Role of sponsor/funder
Introduction	6	Rationale
	7	Objectives
Methods	8	Eligibility criteria
	9	Information sources
	10	Search strategy
	11	Study records (data management; selection process; data collection process)
	12	Data items
	13	Outcomes and prioritization
	14	Risk of bias in individual studies
	15	Data synthesis
Meta-bias(es)	16	Specify plans
Confidence in cumulative evidence	17	How assessed

A published protocol also alerts to the healthcare community that a review is underway providing an audit trail for changes to the protocol. Yet none of the reviewed IJCP articles (Table 1) had published a peer reviewed systematic review protocol.

## Clear review question

The systematic review question will determine the type of studies to be included whether quantitative, qualitative, mixed methods, a review of reviews or a combination.

The review question is often written in a formulaic fashion. For example, “A systematic review of medical and non-medical practitioners’ views of the impact of ehealth” or “Non-medical prescribing versus medical prescribing for acute and chronic disease management in primary and secondary care” [33, 34]. Each includes and demonstrates the use of “PICOS” (P:population; I:intervention; C:comparator; O:outcome; S:setting or study design) [35]. Additions or alternatives include “T” for timeline or “P” as phenomenon of interest. All but one of the identified reviews (Table 1) included a clear review question. The exception varied the focus between evaluation, influence and impact of pharmacist care; each a very different term with clear implications for reporting review findings.

## Independence within reviews

Included in the protocol should be statements about the role of team members in undertaking independent review of the titles, abstracts and full texts, also how any disagreements will be resolved, perhaps by a third member of the team. These steps help to minimise bias and promote transparency of process but in many reviews independence of activity is limited to applying quality criteria thus reducing rigour in the search process, data extraction and reporting aspects. The majority of the rapid review articles (Table 1) make clear statements about author roles but one of the reviews limited independent activity to quality assessment, another review sought to justify lack of independent review by following a narrative review process while a third, a systematic review, did not define team roles.

Common pitfalls of published reviews: lack of a detailed, published protocol to guide all steps of the review; lack of team experience, expertise and independent involvement throughout; lack of independent review or plans for reaching consensus.

## Guidelines and tools for locating, selecting and critically appraising the literature

The search strategy should detail what to look for, how to look for and where to look for (un)published and grey literature (reports, theses, opinion pieces and conference proceedings) plus inclusion/exclusion criteria relevant to the review which will likely include:

Concept mapping

- an important tool in considering alternative search terms, similar to a thesaurus but considering alternative spellings and terms adopted in different countries of practice. Building up search strings as well as understanding the relationships between terms can be eased by working with an experienced team [36];

MeSH (Medical Subject Headings)

- are also helpful in identifying keywords to be combined using Boolean AND/OR/NOT, truncation and wildcard alternatives specific to each database or portal [37];

Language and date inclusions

- perhaps linked to the publication of a seminal text, event or paper identified during the research process or a previous relevant systematic review but must be fully evidenced and justified;

Recording the activity

- of locating and selecting studies using, for example, a PRISMA flowchart alongside spreadsheets to record inclusions/exclusions with reasons for exclusion. The selection process starts with at least two reviewers independently screening titles for inclusion, then abstracts followed by full texts of studies noting, “if in doubt keep it in” [38];

Critical appraisal

- choosing and applying tools relevant to the type of studies identified, considering the unit of analysis (patient, control or study group), quality of the articles and potential for data synthesis: do identified studies use the same unit of analysis or the same elements of PICOS/T?; do studies meet quality criteria or are there so few studies that quality or study design is less relevant? Commonly used tools include those provided by the Cochrane Collaboration, Joanna Briggs Institute (JBI), CASP (Critical Appraisal Skills Partnership) and CEBM (Centre for Evidenced Based Medicine), all offering checklists aligned to study design [1, 28, 35, 39];

Data extraction

- taking meaningful and relevant data from included studies is important in the systematic review process. Data extraction tools should be developed by the review team and piloted against samples of included literature;
- designing or adapting existing tools, plans for dealing with missing data, or applying subgroup analyses using dedicated software (for example, Cochrane’s RevMan; JBI’s Sumari and Connect +) available to authors, consideration of assessment of risk of bias, assessment of heterogeneity (difference in units of analysis which may prevent synthesis), sensitivity or specificity analyses (ability of the search to identify a breadth of studies which are of direct relevance) and, where appropriate, measures of treatment effect [1, 28]. Most importantly, all the evidence indicates that data extraction should be conducted by at least two researchers operating independently or following the option of blinding;

Reducing bias in cumulative evidence

- systematic reviews adopt a robust and rigorous approach aimed at reducing bias and errors during the identification, quality assessment and synthesis (possibly statistical combination) of relevant studies [1]. Bias is the ever present ‘devil in the detail’ with the potential to undermine the quality of any review [25, 26]. It takes many forms, for example selection, omission, commission, source, availability and reporting bias in sourcing, including and excluding articles from a review;
- bias can affect the quality of primary data of an article considered for inclusion in a review. Sampling errors, construct validity of variables, bias in the effect size undermine the intended objectivity. Systemic error through bias introduced into any phase of research, including study design, data collection, synthesis, analysis and publication may skew reported outcomes [25, 26];
- minimising bias is often aided by the application of recognised, standardised tools but seven of the 17 review articles (Table 1), including three systematic reviews, did not report the use of standardised tools.

Common pitfalls of published reviews: lack of focus in the research question(s), bias, drifting from the primary outcome, lack of team experience and subject/methodological expertise.

## Discussion

Many of the evidence-based industry sites have links to frequently asked questions (FAQs), online training and encouragement to conduct systematic reviews including templates, checklists and software for building reports. Online and face-to-face training options are readily available and are strongly recommended for anyone considering undertaking a systematic review. Despite the ready availability and broad range of tools, the 17 reviewed articles (Table 1) demonstrate use of only a limited range of those tools and only applied to limited parts of the review process.

Common pitfalls of published reviews: time spent developing original tools but conversely using tools not adapted to purpose, lack of independent review within the team which is especially an issue where systematic reviews are undertaken as part of an academic activity (student-led review may have limited supervisor input).

## The “must do” list for systematic reviews

There are many guidelines and checklists available from which to create a “must do” list for systematic reviews [1, 4, 24, 27–30, 32, 38–44]. When their elements are mapped, there are clear commonalities, reducing the complexity for would be reviewers:

- a systematic review question (covering, where relevant, PICOS/T)
- background justifying the case for a systematic review
- a review team (expertise and experience in subject area and in conducting systematic reviews)
- a systematic review protocol (potentially registered/published)
- a search strategy (including search terms, inclusion and exclusion criteria, study design, databases and grey literature)
- duplicate, independent critical appraisal
- duplicate, independent data extraction
- synthesis of data (method of combining results, subgroup analysis)
- reporting template and/or checklists (key findings, validity, potential biases, future agenda for research).

## Limitations

This rapid review was limited to the database of one journal over a period of 6 years. Although the review was conducted systematically, it is not presented as a systematic review.

## Conclusion

This rapid review article posed the question, ‘how systematic is systematic?’, before identifying uses and types of reviews, and key factors which limit claims to be systematic. The burgeoning evidence industry offers the tools and guidelines—some of which are described in Table 3-required to conduct systematic reviews, and other types of review, systematically. However, as a research community we have yet to fully engage and so avoid the common pitfalls. Therefore the question remains, not just of IJCP published reviews but potentially all published reviews, ‘how systematic is systematic?’

**Table 3:** A selection of widely recognised resources (guidance, checklists and tools) designed to promote quality in systematic reviews

Resources: guidance, checklists and tools	Last updated	Purpose	Critique
*AMSTAR* a measurement tool to assess systematic reviews	2007	Eleven item online and printer friendly tool with comprehensive guidance to calculate quality and risk of bias	Currently under review aiming to improve on original validated form. Addition tool AMSTAR-NRS (non-randomised studies) under development
*CASP* Critical Appraisal Skills Programme	2006	Wide range of educational resources, tools and checklists to aid critical appraisal of papers identified for review	Public Health Resource Unit provides an accessible set of tools and training options to help all stakeholders ‘make sense of the evidence’. Widely used and recommended by academics and educators
*CEBM* Centre for Evidence Based Medicine	2016	Primarily aimed at medics and medical students, provides education and training, recommended reading as well as checklists and tools and CATMaker (critically appraised topics)	Evidence Based Medicine and Baking offers a novel demonstration of the hierarchy of evidence as a sponge cake but also available as a colourful powerpoint. Accessibility extends to multilingual translations of critical appraisal tools and worksheets
*Cochrane Handbook* for Systematic Reviews of Interventions	2011	Comprehensive guidance and support from expert community of special interest groups. Involves a commitment to maintaining and updating	Earned its reputation to be considered the gold standard guidance for systematic reviews of interventions. Excellent peer review process. Extensive online training for authors including use of RevMan
*CRD Prospero* Centre for Review and Dissemination Guidance for Undertaking Reviews in Health Care including PROSPERO (International Prospective Register of Systematic Reviews)	2009	Open access register of reviews aiming to reduce duplication of effort amongst the international healthcare community	Rapid review prior to acceptance on to the register. Provides an open access audit trail of review progress. Although the protocols are detailed, they are also readily accessible and ‘write-able’
*EQUATOR* Enhancing the QUAlity and Transparency Of health Research	2007	Range of widely recognised tools to ‘promote, teach and practice accurate, complete and ethical publication of health research’	Includes COREQ Consolidated criteria for reporting qualitative research 32-item checklist for interviews and focus groups and STROBE 22-item checklist for Strengthening the Reporting of Observational Studies in Epidemiology. Also available in Spanish
*JBI* Joanna Briggs Institute Systematic review resource package	2014	Aims to guide consistency in conducting and reporting healthcare reviews	Would be reviewers must complete a full set of training modules including use of templating software Sumari and CReMS. Excellent support for protocol development but data extraction software can feel less than transparent to novice reviewers
*PRISMA Statement* Preferred Reporting Items for Systematic Reviews and Meta-Analyses	2009	Reporting checklist and flow diagram for transparency of process available in 8 languages	Detailed and widely used checklist of 27 items and flow diagram. Useful explanatory paper published
*PRISMA-P* Preferred Reporting Items for Systematic Reviews and Meta-Analyses Protocols	2015	Protocol checklist for completeness and transparency of process	Comprehensive with 17 items based on CDSR, PROSPERO, AHRQ. Useful explanatory paper published. Also in 2015, *PRISMA-IPD* (individual patient data) and *PRISMA-NMA* (network meta-analyses)

## Footnotes

1 DARE (Database of Abstracts of Reviews of Effects); CDSR (Cochrane Database of Systematic Reviews); NICE (National Institute for Health and Clinical Excellence); NIHR HTA (National Institute for Healthcare Research Health Technology Assessment); EPPI (Evidence for Policy and Practice Information) Centre; NGC (National Guidelines Clearinghouse; SIGN (Scottish Intercollegiate Guidelines Network).
